# Seoritae Extract Reduces Prostate Weight and Suppresses Prostate Cell Proliferation in a Rat Model of Benign Prostate Hyperplasia

**DOI:** 10.1155/2014/475876

**Published:** 2014-02-19

**Authors:** Hoon Jang, Woong-Jin Bae, Seung-Mo Yuk, Dong-Seok Han, U-Syn Ha, Seong-Yeon Hwang, Shin-Hee Yoon, Sae-Woong Kim, Chang-Hee Han

**Affiliations:** ^1^Department of Urology, The Catholic University of Korea College of Medicine, Seoul St. Mary's Hospital, 222 Banpo-daero, Seocho-Gu, Seoul 137-701, Republic of Korea; ^2^Korea Bio Medical Science Institute, Prime Tower 1st Floor Sinmunno 2-ga, Jongno-gu, Seoul 110-062, Republic of Korea; ^3^The Catholic Agro-Medical Center, The Catholic University of Korea, Seoul St. Mary's Hospital, 222 Banpo-daero, Seocho-Gu, Seoul 137-701, Republic of Korea; ^4^The Catholic University of Korea, Uijeongbu, St. Mary's Hospital 271, Cheon Bo-Ro, Uijeongbu-Si, Gyeonggi-Do 480-717, Republic of Korea

## Abstract

Seoritae is a type of black soybean that is known to have health-promoting effects due to its high isoflavone and anthocyanin contents. We evaluated whether Seoritae extract (SE) had beneficial effects on the reduction of prostate weight in a rat model of benign prostatic hyperplasia (BPH). BPH was induced by intramuscular injections of testosterone enanthate once a week for 5 weeks in Sprague-Dawley rats, and rats were treated with or without daily oral doses of SE during BPH induction. After 5 weeks, the oxidative stress (superoxide dismutase and 8-hydroxy-2-deoxyguanosine), apoptosis (caspase-3), and activity of 5-alpha reductase were evaluated in the serum and prostate. The SE treatment group showed a significant decrease in prostate weight, oxidative stress, apoptosis, and 5-alpha reductase activity compared to the nontreated BPH group. These results show that SE is effective in decreasing the weight and proliferation of the prostate, and suggest that SE may be an effective treatment for BPH.

## 1. Introduction

The average life-span of human beings is gradually increasing due to development in science and medicine [1]. As a result, the elderly currently account for a significant portion of the population, and the quality of life in this age group has become a social concern. Benign prostate hyperplasia (BPH), which is the limitless growth of the prostate without malignancy, has been shown to have a high incidence in elderly males and affects their quality of life.

Although the molecular biological mechanisms influencing the etiology of BPH have not been elucidated; dihydrotestosterone (DHT), which is the more active from of testosterone and is converted from testosterone by 5-alpha reductase, is known to play a central role in the prostate growth [2] and the oxidative stress-mediated mechanism is believed to also be associated with prostate cell hyperproliferation and tissue deformity [3, 4]. In our previous report, we suggested that the oxidative stress mechanism is related to the occurrence and progression of BPH and that anthocyanin is a potent antioxidant that can decrease prostate volume and prevent BPH progression [5]. This study builds upon the work from our previous study by evaluating the relationship between oxidative stress and the occurrence and progression of BPH. In addition, we hypothesized that inhibition of 5-alpha reductase would decrease the conversion of DHT from testosterone and antioxidant reactions that serve to reduce oxidative stress may prevent the occurrence and progression of BPH.

Seoritae is a type of black soybean (*Glycine max* (L.) Merr.) grown in Korea, which gets its name from the fact that it is harvested early in October when the first frost occurs. Unlike other black soybeans, the inside of Seoritae is of a bluish color. It is a traditional Korean food and is known to have health-promoting effects due to its high isoflavone and anthocyanin contents. Isoflavones have variable effects on growth factor inhibition [6], antioxidant properties [7, 8], cell adhesion [9], 5-alpha reductase activity [10, 11], and angiogenesis [12].

Anthocyanin is a known antioxidant that has antiangiogenic, anticarcinogenic, and antioxidant effects [13–15]. In particular, we hypothesized that the 5-alpha reductase inhibitory effect as well as the antioxidant properties of the isoflavones and anthocyanin in SE would be helpful in preventing the occurrence and progression of BPH.

Therefore, we administered Seoritae extract (SE) to rats during the induction of BPH. Prostate weight, oxidative stress, apoptosis, and 5-alpha reductase activity were then measured in order to investigate the mechanism by which SE regulates oxidative stress and prostate cell proliferation so as to determine its potential in treating BPH.

## 2. Materials and Methods

### 2.1. Preparation of SE

The SE used in our experiment was produced using the following method: Seoritae (1500 g) samples were extracted with 12,000 mL of 30% ethanol for 3 h at 90–100°C. The solution was then filtered twice through a 50 *μ*m and a 1 *μ*m filter and concentrated in a vacuum evaporator (60°C) to 70 brix. The residual solvent was removed from SE by using a drying machine for 18 h at 60°C in a vacuum. The resulting powder was then stored in plastic bag until use.

### 2.2. Analysis of Isoflavones and Anthocyanin from Seoritae Extracts

**Figure 1:**
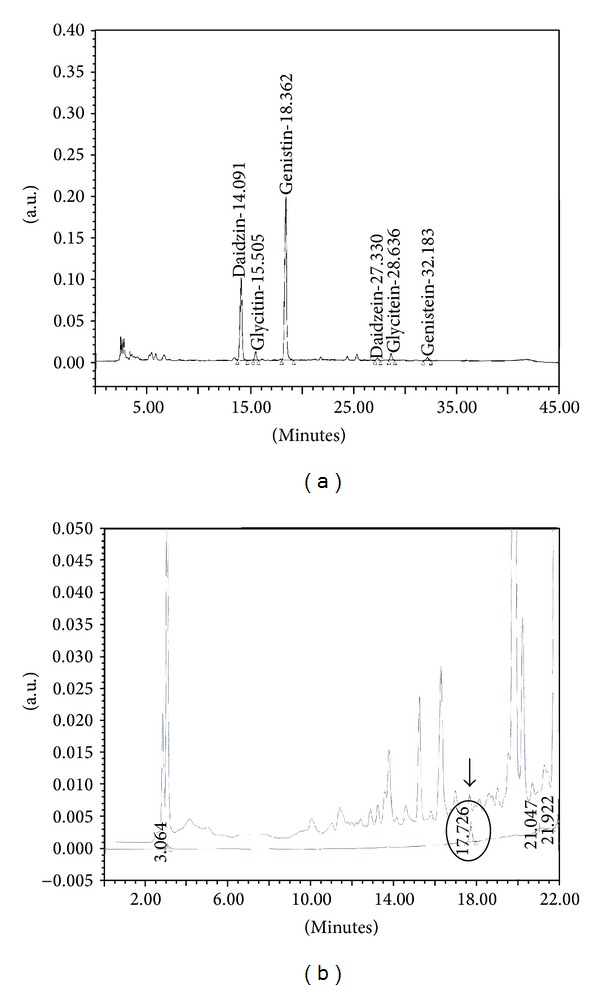
HPLC chromatogram of the Seoritae extract. (a) Six peaks were obtained and each peak represented the isoflavone content. (b) The peak with the circle indicates Cyanidin-3-*O*-glucoside in standard compounds. A corresponding peak was seen in the SE HPLC chromatogram (black arrow).

Isoflavones in SE were analyzed by high-performance liquid chromatography (HPLC) using a Waters 2695 Preparation Module HPLC system with a Waters 996 Photodiode Array Detector (Waters Corporation; Milford, MA, USA). Six peaks were obtained in the HPLC chromatogram by diode array detection (DAD) at 260 nm. Major peaks 1, 2, 3, 4, 5, and 6 were identified as daidzin, glycitin, genistin, daidzein, glycitein, and genistein, respectively, by comparison with HPLC retention times of our standard compounds (Figure 1(a)).

The anthocyanin content in SE was analyzed by HPLC using a Waters HPLC system with a 2487 Dual Wavelength Detector set at 520 nm. Cyanidin-3-*O*-glucoside, one of the anthocyanins, was identified in the HPLC chromatogram by comparison with the HPLC retention times of our standard compounds (Figure 1(b)). The proportions of the isoflavones and anthocyanins in the SE are shown in Table 1.

### 2.3. Animal Groups and Treatment Protocol

Forty-eight 16-week-old Sprague-Dawley male rats were treated under a protocol approved by the Institutional Animal Care and Use Committee (CUMC-2013-0117-01) and handled according to National Institutes of Health (NIH) guidelines. Rats were divided equally into 4 groups (*n* = 12 each): control, BPH, and BPH treated with SEs (BPH + SE1 and BPH + SE2). To prevent the influence of intrinsic testosterone, all rats in the BPH and BPH + SE groups underwent bilateral orchiectomies performed 3 days prior to the induction of BPH. Prostate hyperplasia was induced in the BPH and BPH + SEs groups once a week by intramuscular injections of testosterone enanthate (25 mg; Rotexmedica GmbH; Trittau, Germany) for 5 weeks. During BPH induction, rats in the BPH + SEs groups were treated daily with oral SE at two concentrations (the BPH + SE1 group received 228 mg/kg of SE and the BPH + SE2 group received 457 mg/kg of SE), which were dissolved in 1 mL distilled water and administered orally through an 8F red Rob-Nel catheter once a day for 5 weeks. The SE2 dose was the converted dosage for rats that represented the maximal suggested dosage of isoflavones for humans under the Korean Ministry of Food and Drug Safety (MFDS) guidelines. After 5 weeks, all rats were sacrificed. The blood was collected from the vena cava and the prostate was removed and weighed. The oxidative stress and the activity of 5-alpha reductase were analyzed in the serum and prostate, and apoptosis was assessed in the prostate samples.

### 2.4. Measurement of Oxidative Stress in Serum

To evaluate the oxidative stress in serum, we measured the total activity of superoxide dismutase (SOD). The total activity of SOD in serum was determined using a WST SOD assay kit (Dojindo Molecular Technologies; Kumamoto, Japan), which estimates SOD activity by measuring the inhibition of xanthin oxidase activity. Serum preparation for the total activity of SOD was performed according to the manufacturer's protocol. The optical density (OD) was determined at 450 nm using a microplate reader (Bio-Rad Model 550; CA, USA). The SOD-like activity was calculated by using the following equation: SOD activity = {[(A  blank1 − A  blank3) − (A  sample − A  blank2)]/(A  blank1 − A  blank3)} × 100.

### 2.5. Measurement of Oxidative Stress in the Prostate

Oxidative stress in the prostate was evaluated by quantifying the levels of 8-hydroxy-2-deoxyguanosine (8-OHdG) as a measurement of oxidatively modified DNA. Total DNA was extracted from the tissues using a DNeasy Blood and Tissue Kit (Qiagen, Valencia, CA), according to the manufacturer's instructions. The levels of 8-OHdG were measured using a DNA oxidation kit (Highly Sensitive 8-OHdG Check ELISA; Japan Institute for the Control of Aging; Fukuroi, Japan), according to the manufacturer's protocol. The 8-OHdG standard (0.5–40 ng/mL) or 15–20 *μ*g of DNA purified from the tissues was incubated for 1 h with a monoclonal antibody against 8-OHdG in a microtiter plate precoated with 8-OHdG. After the color was developed with the addition of 3,3′,5,5′-tetramethylbenzidine, absorbance was measured at 450 nm. Tissue sample concentration was calculated from a standard curve and was corrected for DNA concentration.

### 2.6. Measurement of Apoptosis

To assess apoptosis in the prostate, the concentration of caspase-3 was measured using an ApoTarget Caspase-3/CPP32 Colorimetric Protease Assay Kit (Invitrogen; Camarillo, CA, USA). Tissue preparation for the quantification of caspase-3 was performed according to the manufacturer's protocol. Samples were read at 400 nm in a microplate reader.

### 2.7. Measurement of 5-Alpha Reductase Activity

The activity of 5-alpha reductase was evaluated by the quantitative measurement of steroid 5-alpha reductase 2 (SRD5a2). In serum and prostate tissue, the SRD5a2 levels were measured in duplicate with SEM285Ra (USCN; Houston, TX, USA), a commercially available rat-specific enzyme-linked immunosorbent assay (ELISA) kit. Serum and tissue preparation was performed according to the manufacturer's protocol. Samples were read at 450 nm in a microplate reader.

### 2.8. Statistical Analysis

Data were analyzed statistically and expressed as the mean ± SD. Groups were compared using ANOVA followed by Tukey's test for multiple comparisons. The level of significance was set at *P* < 0.05.

## 3. Results

### 3.1. Prostate Weight of the BPH and BPH + SE Groups

The mean prostate weight of the BPH group was significantly higher than that of the control group (*P* < 0.05). Compared with the BPH groups, administration of SE led to significant reduction in prostate weight (*P* < 0.05) (Table 2). The reduction in prostate weight was proportional to the increase in the dose of SE applied.

### 3.2. Pathohistological Findings in Each Experimental Group

**Figure 2:**
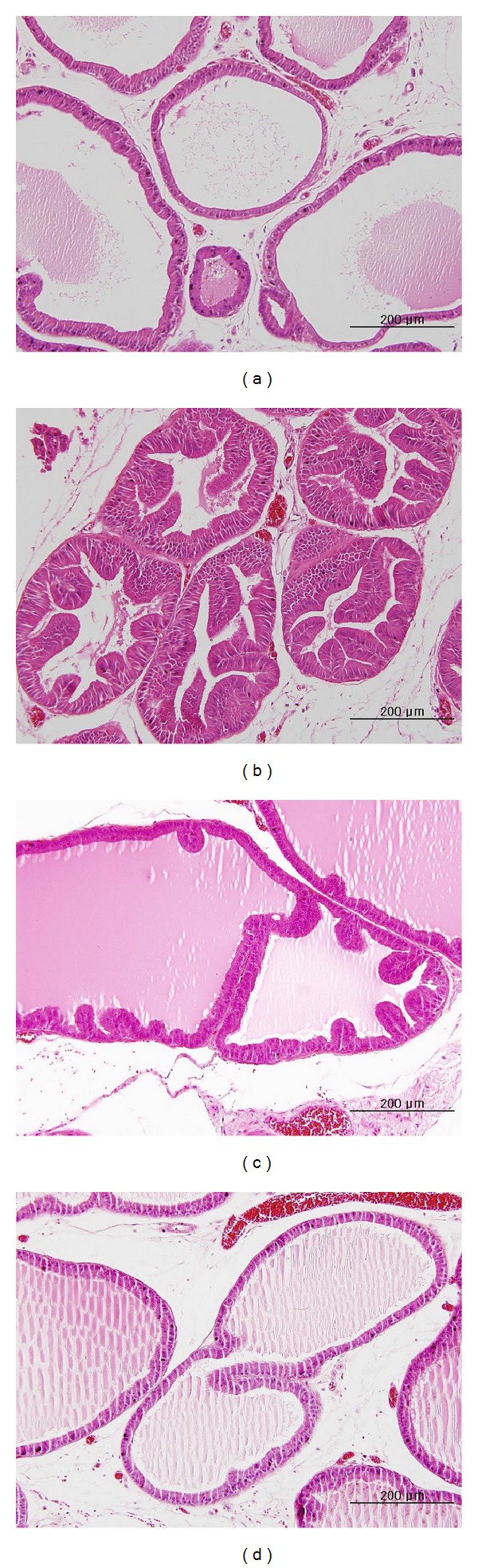
Histological analysis of prostate specimens taken from each experimental group. (a) Secretory luminal cells were lined with a single layer of low columnar epithelium and the acini were filled with pale eosinophilic materials (H & E stain, ×200). (b) The epithelial cells in glands were arranged as several uneven layers and the gland was excessively developed (H & E stain, ×200). (c) In comparison to (b), the proliferation of columnar epithelial cells in the BPH + SE1 group was restricted, and the development of the gland was limited (H & E stain, ×200). (d) It is difficult to find a difference between (d) and (a), except for the multiple layers of columnar epithelial cells in some parts of the gland in (d). H & E: hematoxylin and eosin; (a) control; (b) BPH; (c) BPH + SE1; (d) BPH + SE2.

In the control group, one layer of low-columnar epithelial cells formed a secretory lumen that was filled with thin acidophilic materials. Undeveloped epithelial cells forming the prostate gland were arranged as a single layer (Figure 2(a)). In contrast, the epithelial cells in the BPH group were arranged in several uneven layers, and the gland was excessively developed (Figure 2(b)). In the BPH + SE1 group, columnar epithelial cells were arranged as multiple layers, and the proliferation of epithelial cells and the number of glands were increased compared with the normal group. However, in comparison with the BPH group, prostate cell proliferation and the development of glands were noticeably decreased (Figure 2(c)). It was difficult to find a histological difference between the control group and the BPH + SE2 group (Figure 2(d)).

### 3.3. Comparison of Oxidative Stress in the Serum and Prostate

Oxidative stress was assessed by measurement of SOD activity in the serum as well as the level of 8-OHdG in prostate tissues. A significant increase in oxidative stress was found in the BPH group compared with the control group (*P* < 0.05). In the two BPH + SE groups, however, oxidative stress was significantly reduced compared to the BPH group (*P* < 0.05) (Table 2). The reduction of oxidative stress was proportional to the increase in the dose of SE applied.

### 3.4. Comparison of Apoptosis

A significant increase in the concentration of caspase-3 was found in the BPH group when compared with the control group (*P* < 0.05). In the BPH + SE groups, however, the concentration of caspase-3 was significantly reduced compared to the BPH group (*P* < 0.05) and significantly increased compared with the control group (*P* < 0.05) (Table 2).

### 3.5. Comparison of 5-Alpha Reductase Activity in the Serum and Prostate Tissues

A significant increase in 5-alpha reductase activity was found in the serum and prostate samples from the BPH group when compared to the control group (*P* < 0.05). On the other hand, in the BPH + SE groups, the activity of 5-alpha reductase was reduced in comparison to the BPH group (*P* < 0.05) (Table 2). However, the reduction in 5-alpha reductase activity was not proportional to the increase in SE dose. There was no significant difference in the 5-alpha reductase activity in the sera taken from the BPH + SE groups (*P* > 0.05). In prostate tissue, the BPH + SE1 group showed an increase in 5-alpha reductase activity in comparison to the BPH + SE2 groups (*P* < 0.05).

## 4. Discussion

The molecular biological mechanisms influencing the etiology of BPH have not been elucidated; however, BPH has a high incidence among elderly males [16], and aging is known to be a risk factor for developing BPH [17]. The free radical theory suggests that free radicals generated in the human body during the process of producing energy lead to oxidative injury of cell components such as proteins, DNA, and lipids. This results in the loss of tissue and organ functions as well as cell death [18, 19] and is related to the occurrence and progression of BPH. In fact, a correlation between oxidative stress and BPH occurrence has been identified in several studies [3, 4], and we therefore hypothesized that the oxidative stress mechanism is related to the occurrence and progression of BPH. Moreover, anthocyanin, as a potent antioxidant, was found to be effective in decreasing prostate volume and preventing the progression of BPH in our previous report [5]. In addition, the presence of testes producing androgens is a known risk factor for BPH since androgens, including testosterone and dihydrotestosterone (DHT), play a central role in regulating cell proliferation and death in the prostate. In particular, the expression of DHT, which is the most active form of testosterone that is converted from testosterone by 5-alpha reductase, is correlated with the occurrence and progression of BPH [2].

Considering the above, we hypothesized that treatment with a compound that inhibits the conversion of testosterone to DHT and decreases oxidative stress would prevent the occurrence and progression of BPH. The main findings of the present study were as follows: (1) BPH was induced by injection of testosterone enanthate, and prostate weight was decreased as a result of the SE treatment; (2) oxidative stress in the serum and the prostate was increased by induction of BPH and was reduced by SE treatment; (3) expression of caspase-3, which is a measure of apoptosis, was increased in rats with BPH and decreased by the SE treatment; (4) the activity of 5-alpha-reductase was increased due to the induction of BPH but was decreased as a result of the SE supplementation.

In our study, the HPLC chromatogram revealed that SE was composed of variable isoflavones and anthocyanins (Table 1). Therefore, the effect of SE was based on the properties of these isoflavones and anthocyanins. Based on our results, we hypothesized that the occurrence and progression of BPH were related to the activity of 5-alpha reductase and the cellular damage by oxidative stress. We also suggest that the reduction in prostate weight after the administration of SE might be due to the inhibitory effect of 5-alpha reductase as well as the antioxidant properties of the isoflavones and anthocyanin in SE.

Oxidative stress reflects an imbalance between the systemic manifestation of reactive oxygen species (ROS) and a biological system's ability to readily detoxify the reactive intermediates or to repair the resulting damage. Disturbances in the normal redox state of cells can cause toxic effects through the production of peroxides and free radicals that damage all components of the cell including proteins, lipids, and DNA. This is thought to result in the loss of cellular and tissue function.

In our study, oxidative stress was increased in the serum and prostate as a result of BPH, which was confirmed by measuring the activity of SOD in the serum and the level of 8-OHdG in the prostate. In addition, these changes were reversed after the administration of SE. Increased oxidative stress might cause oxidative injury to various cell cycle control proteins and disrupt the balance between proliferation and cell death in the prostate, resulting in the occurrence and progression of BPH.

It is known that genistein and daidzein, two types of isoflavones, inhibit hydrogen peroxide production and superoxide anion generation in cells, possibly via indirect regulation of antioxidant enzyme levels and isoflavones. Reduced derivatives of these compounds can also inhibit microsomal lipid peroxidation in vitro [7, 20]. Furthermore, anthocyanin leads to the removal of superoxide, singlet oxygen, hydrogen peroxide, and hydroxyl radicals and also stabilizes and inactivates free radicals and prevents cellular oxidative stress [21].

We suggest that the improvement of oxidative stress after the administration of SE in our study was due to the antioxidant effects of the isoflavones and anthocyanins. This effect might have decreased the oxidative injury to the prostate cells and prevented abnormal cell proliferation, thus restoring the balance between proliferation and apoptosis in BPH.

Interestingly, the BPH group showed a markedly increased concentration of caspase-3, the final effector of cell lysis, compared to that observed in the control group. Similar results were reported in several studies [22–25]. Kosova et al. reported that a considerable degree of oxidative stress and DNA damage occurs in BPH (although less than that occurring in prostate cancer) and that levels of caspase-3 are higher in BPH than in prostate cancer [25]. In addition, Shariat et al. reported that levels of Bcl-2, Bax, Ki-67, and caspase-3 were highly elevated in patients with BPH when compared to patients with a normal prostate [24]. According to the above reports and our result, we suggest that elevated caspase-3 in BPH might be a homeostatic reaction that balances the apoptosis-proliferation equilibrium, which occurs due to abnormal hyperproliferation of prostate cells induced by DNA damage from oxidative stress.

In contrast, the concentration of caspase-3 in BPH + SE groups was markedly increased compared with that of the control group and was significantly decreased compared with that of the BPH group. We suggest that the number of cells involved in apoptosis in BPH + SE groups may have been decreased compared to that in the BPH group. This is because cellular damage from oxidative stress, which leads to abnormal prostate cell hyperproliferation, might be decreased by antioxidant effect of SE. Thus, the homeostatic reaction that balances the apoptosis-proliferation equilibrium in the BPH + SE groups might have been decreased relatively to the BPH group. This might have resulted in a significant decrease in the concentration of caspase-3 in BPH + SE groups when compared to the BPH group.

It is well known that isoflavones have the ability to block the overproduction of certain steroid hormones that influence the promotion and progression of BPH. Specifically, isoflavones are known to inhibit 5-alpha reductase, aromatase, and 17 beta-hydroxysteroid dehydrogenase enzymes, which block the synthesis of DHT, estrone, and other steroid hormones, respectively. DHT is known to enhance prostate cell division and is directly linked to the promotion and progression of BPH [2, 10, 26]. In our study, 5-alpha reductase activity was increased in the BPH group and decreased after administration of SE. These results demonstrated that isoflavones inhibit 5-alpha reductase and suggest that this inhibitory effect may be one of the main mechanisms by which SE decreases prostate weight and prevents the progression of BPH.

We note that our study has several limitations. First, there are many proposed mechanisms by which isoflavones might induce their therapeutic effects, including inhibition of protein tyrosine kinase [6], binding to estrogen receptors [27], inhibition of ROS production [7, 8], inhibition of angiogenesis [28], modulation of sex steroid binding proteins [29], and inhibition of 5 alpha-reductase activity [6]. Among these possibilities, we suggest that the antioxidant effect and the inhibition of 5-alpha reductase are the main mechanisms involved in the SE-induced decrease in prostate weight. However, it is possible that other mechanisms may contribute to the antioxidant effects of isoflavone and suggest that further study on the relevance between these mechanisms and the decrease in the prostate weight after SE administration of SE should be conducted.

Second, our results suggested that the antioxidant effect of anthocyanin contributed to the reduction in prostate weight in this study. It is well established that anthocyanin is a strong antioxidant [21] and effective in reducing prostate weight in an animal model of BPH [5]; however, the anthocyanin content in SE was found to be considerably low. Therefore, the contribution of anthocyanin in relation to our results will need to be assessed further.

## 5. Conclusions

Administration of SE to rats during induction of BPH resulted in a reduction in prostate weight, oxidative stress, and 5-alpha reductase activity, as well as a decrease in apoptosis. We believe that the occurrence and progression of BPH are related to an oxidative stress-mediated mechanism and increasing activity of 5-alpha reductase. Also we believed that the antioxidant properties and inhibitory effect of 5-alpha reductase of isoflavones and anthocyanin in SE would be effective in treating BPH. We therefore suggest that SE has the potential to replace or improve current BPH treatments.

## Figures and Tables

**Table 1:** HPLC analysis of isoflavones and anthocyanin content (mg/g) of Seoritae extract. Triplicate samples of the Seoritae extract were analyzed by HPLC.

Content	Seoritae extract		
Daidzin	3.171	4.146	4.861
Glycitin	0.287	0.369	0.438
Genistin	3.750	4.906	5.726
Daidzein	0.036	0.048	0.064
Glycitein	0.161	0.207	0.240
Genistein	0.043	0.055	0.073
Total	**7.448**	**9.731**	**11.402**

Anthocyanin (Cyanidin-3-O-glucoside)	0.120	0.122	0.124

**Table 2:** Comparison of parameters in each group.

	Prostate weight (g)	Oxidative stress		Apoptosis	Activity of 5-alpha reductase	
		Activity of SOD in serum (%)	Concentration of 8-OHdG in prostate (ng/mL)	Concentration of caspase-3 in prostate (ug/mL)	In serum (%)	In prostate (%)
Control	1.040 ± 0.061	35.761 ± 2.115	0.620 ± 0.061	3.661 ± 0.053	5.668 ± 0.292	7.178 ± 0.092
BPH	1.421 ± 0.039*	81.345 ± 0.892*	7.651 ± 0.306*	24.456 ± 1.625*	18.863 ± 0.648*	27.176 ± 0.719*
BPH + SE1	0.988 ± 0.040**	62.047 ± 0.875**	6.493 ± 0.253**	17.212 ± 0.787**	11.515 ± 0.344**	18.177 ± 0.643**
BPH + SE2	0.878 ± 0.068**	55.816 ± 1.488**	4.060 ± 0.355**	12.975 ± 0.703**	11.347 ± 0.120**	15.453 ± 0.382**

The mean prostate weight of the BPH group was significantly higher than that of the control group, whereas the means of the BPH + SE groups were significantly reduced compared to the BPH group.

A significant increase in oxidative stress was found in the BPH group compared with the control group, and oxidative stress was significantly reduced in the two BPH + SE groups.

A significant increase in the concentration of caspase-3 was found in the BPH group compared with the control group, and it was significantly decreased in the two BPH + SE groups.

A significant increase in 5-alpha reductase activity was found in the BPH group compared with the control group, and it was significantly reduced in the two BPH + SE groups.

BPH: benign prostatic hyperplasia group; BPH + SE1: BPH group treated with Seoritae extract (228 mg/kg); BPH + SE2: BPH group treated with Seoritae extract (457 mg/kg); SOD: superoxide dismutase; 8-OHdG: 8-hydroxy-2-deoxyguanosine.

*Significant difference (*P* < 0.05) compared with the control group.

**Significant difference (*P* < 0.05) compared with the BPH group.
